# *EIF1AX* mutation in thyroid tumors: a retrospective analysis of cytology, histopathology and co-mutation profiles

**DOI:** 10.1186/s40463-022-00594-6

**Published:** 2022-11-12

**Authors:** Noha Elsherbini, Dong Hyun Kim, Richard J. Payne, Thomas Hudson, Véronique-Isabelle Forest, Michael P. Hier, Alexandra E. Payne, Marc P. Pusztaszeri

**Affiliations:** 1 Faculty of Medicine, McGill University, Montreal, QC Canada; 2 Department of Pathology, Sir Mortimer B. Davis-Jewish General Hospital, McGill University, 3755, Chemin de La Côte Ste Catherine, Montreal, QC H3T 1E2 Canada; 3 Department of Otolaryngology - Head and Neck Surgery, Sir Mortimer B. Davis-Jewish General Hospital, McGill University, Montreal, QC Canada; 4 Marianopolis College - Health Sciences, Montreal, QC Canada

**Keywords:** *EIF1AX*, Thyroid nodule, Fine needle aspiration, FNA, Cytology, Molecular testing

## Abstract

**Background:**

The *EIF1AX* mutation has been identified in various benign and malignant thyroid lesions, with a higher prevalence in poorly differentiated thyroid carcinoma (PDTC) and anaplastic thyroid carcinoma, especially when combined with *RAS* or *TP53* mutation. However, data and clinical significance of *EIF1AX* mutations in thyroid nodules is still limited. We investigated the prevalence of *EIF1AX* mutations and co-mutations in cytologically indeterminate thyroid nodules at our institution.

**Methods:**

A 5-year retrospective analysis was performed on surgically resected thyroid nodules with identified *EIF1AX* mutations on molecular testing with ThyroseqV3^®^. Mutation type and presence of co-mutations were correlated with histopathologic diagnosis and clinical characteristics. Histopathology diagnoses were subsequently categorized as benign, borderline, malignant or aggressive malignant (≥ 10% PDTC component). Chi-square test was used to compare the malignancy associations of the: 1) A113_splice mutation compared to non-A113_splice mutations 2) singular A113_splice mutations compared to singular non-A113_splice mutations. Fisher’s Exact Test was used to determine the association of A113_splice mutation with aggressive malignancies compared to non-A113_splice mutations. A *p* value of 0.05 or less was considered statistically significant.

**Results:**

Out of 1583 patients who underwent FNA, 621 had further molecular testing. 31 cases (5%) harbored *EIF1AX* mutations. Of these cases, 12 (38.7%) were malignant, 2 (6.5%) were borderline, and 17 (55%) were benign. 4/31 cases (13%) were aggressive malignant (≥ 10% PDTC component). The most prevalent mutation was the A113_splice mutation at the junction of intron 5 and exon 6 (48%). All other mutations, except one, were located at the N-terminal in exon 2. 7/31 cases (22.6%) harbored ≥ 1 co-mutation(s), including 4 *RAS, 3 TP53, 1 TERT and 1 PIK3CA,* with 86% of them being malignant. All 4 nodules with *RAS* co-mutations were malignant including one PDTC.

**Conclusion:**

Our study reports the largest cohort of *EIF1AX* mutations in Bethesda III/IV FNA samples with surgical follow-up to our knowledge. The presence of the *EIF1AX* mutation confers a 45.2% risk of malignancy (ROM) or borderline after surgery. However, the coexistence of *EIF1AX* mutations with other driver mutations such as *RAS, TERT* or *TP53* conferred an 86% ROM. While 55% of thyroid nodules were benign at the time of surgery, the possible malignant transformation of these nodules, had they not been resected, is unknown. Finally, 13% of the nodules with *EIF1AX* mutations were aggressive with a significant PDTC component. These findings can further aid in clinical decisions for patients with thyroid nodules.

**Graphic Abstract:**

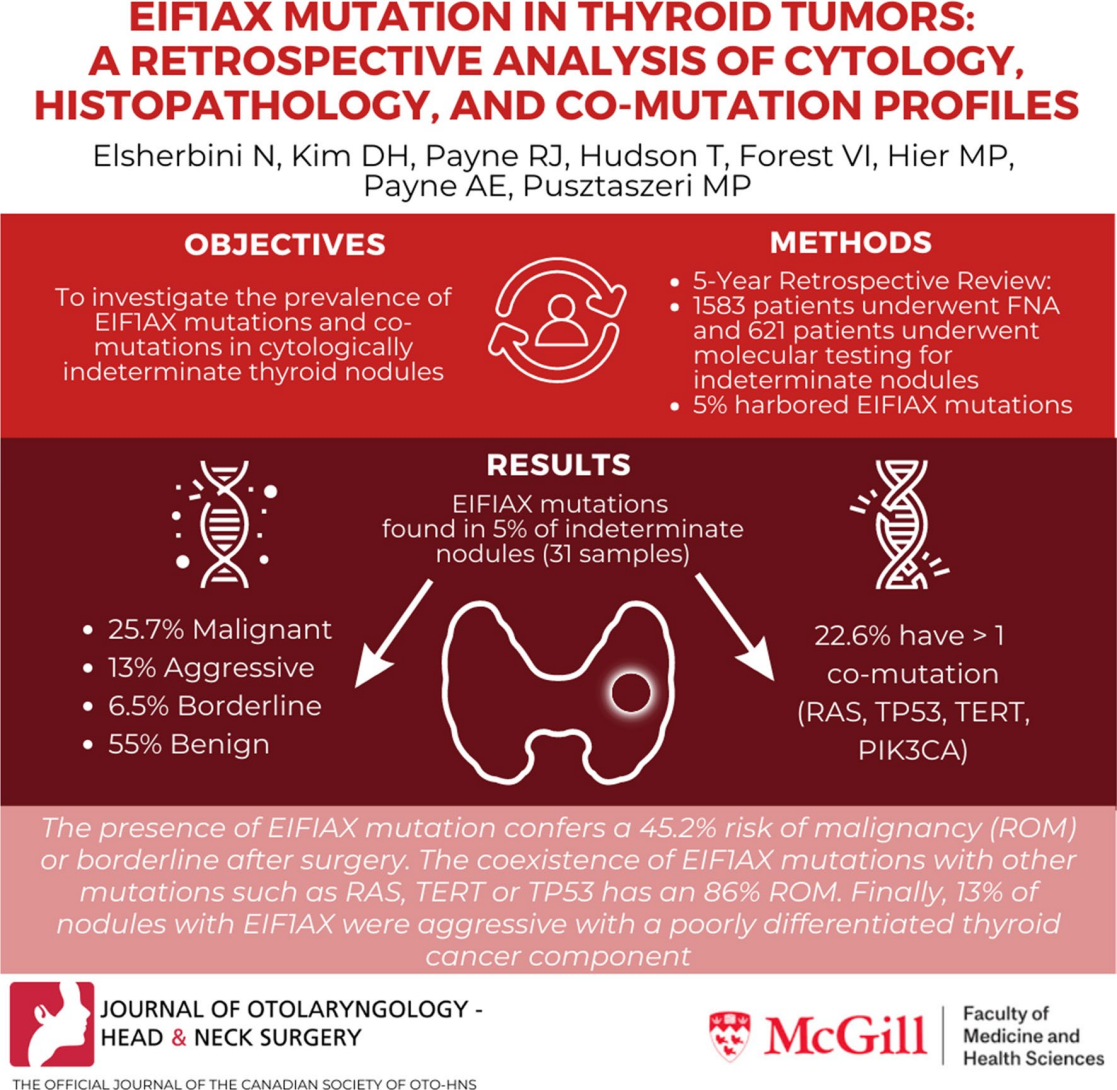

## Introduction

Thyroid cancer is the most common endocrine malignancy, and its incidence has increased three-fold in recent decades in part due to improved detection of indolent lesions by sensitive imaging techniques [1, 2].

Fine needle aspiration (FNA) is a widely used first-line diagnostic approach to differentiate between benign and malignant tumors. However, 15–30% of all thyroid lesions investigated by FNA cytology are indeterminate [3]. The overall risk of cancer is ∼10–30% for atypia of undetermined significance/follicular lesion of undetermined significance (AUS/FLUS) (Bethesda III category) and 25–40% for follicular neoplasm (FN) or suspicious for FN (Bethesda IV category). Surgically resecting all indeterminate lesions will result in 60–70% unnecessary operations. Thus, in addition to cytomorphology, molecular profile testing that elucidates the genetic signatures of thyroid nodules has been paramount in improving diagnosis, prognostication, and management of thyroid lesions.

The Cancer Genome Atlas (TCGA) study was the first landmark study to describe the mutation of *EIF1AX* (eukaryotic translation initiation factor 1A, X-linked) in thyroid tumors, more specifically in papillary thyroid carcinoma (PTC) [1]. Since the TCGA study, the *EIF1AX* mutation has been identified in other thyroid lesions including nodular hyperplasia (NH), follicular adenomas (FA), follicular thyroid carcinoma (FTC), Hürthle cell carcinoma (HCC), poorly-differentiated thyroid carcinoma (PDTC) and anaplastic thyroid carcinoma (ATC). The *EIF1AX* mutation is more frequently identified in PDTC (11%) and ATC (9–25%) compared to PTC (1–2.5%) and FTC (5.1%) [2, 4–6]. Moreover, PDTC with *EIF1AX* mutation reports lower survival rate than PDTC with wild type *EIF1AX* [7].

The most commonly detected *EIF1AX* mutation is the A113_splice mutation at the C-terminal. The other varieties of mutations, mostly missense and one truncating type are located in the N-terminus.

Overall, data on the prevalence and phenotypic correlations of *EIF1AX* mutations in thyroid nodules is still limited. As a result, the clinical significance of finding *EIF1AX* mutations in isolation or combined with other mutations in FNAs is uncertain. The aim of this study was to determine the prevalence, the risk of malignancy and the histopathological outcome of *EIF1AX* mutations in a series of FNA specimens from indeterminate thyroid nodules (Bethesda III and IV) with surgical follow-up.

## Materials and methods

### Study design and ethics

The study retrospectively analyzed a cohort of patients who had undergone thyroid nodule examination by FNA and molecular profiling prior to surgery. Ethics approval for the study (MP-37-2020-5791), was obtained from the Research Ethics Board of the Jewish General Hospital and of the McGill University Health Center, both in Montreal, Quebec.

### Patient selection

Patients above 18 years old from the two hospital sites who underwent thyroid FNA between January 2016 to September 2020 with indeterminate cytology (Bethesda III and IV) were screened for molecular profiling using Thyroseq V3 for molecular profiling using Thyroseq V3^®^. All surgical patients found to have *EIF1AX* mutation in the molecular profiling were included in this present study.

### Sample collection

Following patient consent and counselling, two otolaryngology surgeons trained in thyroid surgery performed ultrasound guided FNAs. FNA cytology and Bethesda scores were assigned by pathologists using the Bethesda system for reporting thyroid cytopathology [8]. Patients with indeterminate cytology (Bethesda III and IV) were informed and counseled on the risks, benefits and cost of the management options including diagnostic surgery, regular surveillance, or molecular testing. For those who proceeded with the molecular testing, FNA specimen was collected in the ThyroSeqPreserve solution and sent to the Molecular & Genomic Pathology Lab in Pittsburgh, Pennsylvania via courier. Thyroidectomies were performed where appropriate. In general, when an *EIF1AX* mutation was found, the patient was counseled towards a more conservative surgery and not a total thyroidectomy given the low likelihood of aggressive disease. This differs from the counseling that occurred when more aggressive molecular mutations/alterations were found such as a *BRAF V600E* mutation, *RET* fusion, or *NTRK3* fusion. The surgical approach was not altered based on whether the *EIF1AX* mutation was splice or non-splice as the findings in this study were not yet realized. The resected specimens were examined with the tumors classified according to the 2017 WHO classification of thyroid tumors [9]. One pathologist specializing in thyroid pathology reviewed the final histopathology findings to ensure uniform assessments.

### Data collection

The following information was collected from each patient in the analyses: age, sex, location of the tumor (left/right/isthmus), Bethesda scores, date of the operation, final pathology diagnosis, *EIF1AX* mutation type and location, and co-mutations [9]. Further characterization of the different aggressivity of tumors (benign, borderline, malignant-low risk and aggressive-malignant) was done based on histology [10]. Benign included FA and hyperplastic/adenomatoid nodule(s). Borderline included non-invasive follicular thyroid neoplasm with papillary-like nuclear features (NIFTP) and well-differentiated follicular neoplasm of uncertain malignant potential (WDFTUMP). Malignant-low-risk included PTC and minimally invasive FTCs or HCCs. Malignant-aggressive included PDTC cases and any well-differentiated carcinoma (PTC, FTC, HCC) with a PDTC component > 10%, as defined by the Turin criteria [9, 10]. Incidental papillary thyroid microcarcinomas and other thyroid neoplasms that were not consistent with the thyroid nodule sampled by FNA were excluded from the analysis.

### Statistical analysis

Clinical data collected were tabulated using IBM SPSS Statistics Version 27™. Chi-square test was used to compare the malignancy associations of the: (1) A113_splice mutation compared to non-A113_splice mutations and (2) singular A113_splice mutations compared to singular non-A113_splice mutations. Fisher’s Exact Test was used to determine the association of A113_splice mutation with aggressive malignancies compared to non-A113_splice mutations. Benign and borderline pathologies were grouped together for the purposes of statistical analyses as the clinical outcome of the histopathology were the same (i.e., surgical resection). A p-value of 0.05 or less was considered statistically significant.

## Results

A total of 1583 patients who underwent thyroidectomies (total or subtotal) from January 2016 to September 2020 inclusively were screened. Of these, 621 patients underwent molecular profiling. 31 tumors were found to have the *EIF1AX* mutation, resulting in an overall mutation frequency of 4.99% in Bethesda III/IV categories. The average age of patients harboring the mutation was 62 years old (range 43–88), and 25 (81%) were female. Patient characteristics are presented in Table 1.

**Table 1:** Demographics of the patients with *EIF1AX* mutated thyroid nodules

Demographics	
Mean age, years (Standard Deviation)	62 (12)
Female sex, n (%)	25 (81%)
Average U/S size, cm (Standard Deviation)	2.7 (1.3)

Cytopathological diagnosis	
Bethesda III, n (%)	21 (68%)
Bethesda IV, n (%)	10 (32%)

**Fig. 1:**
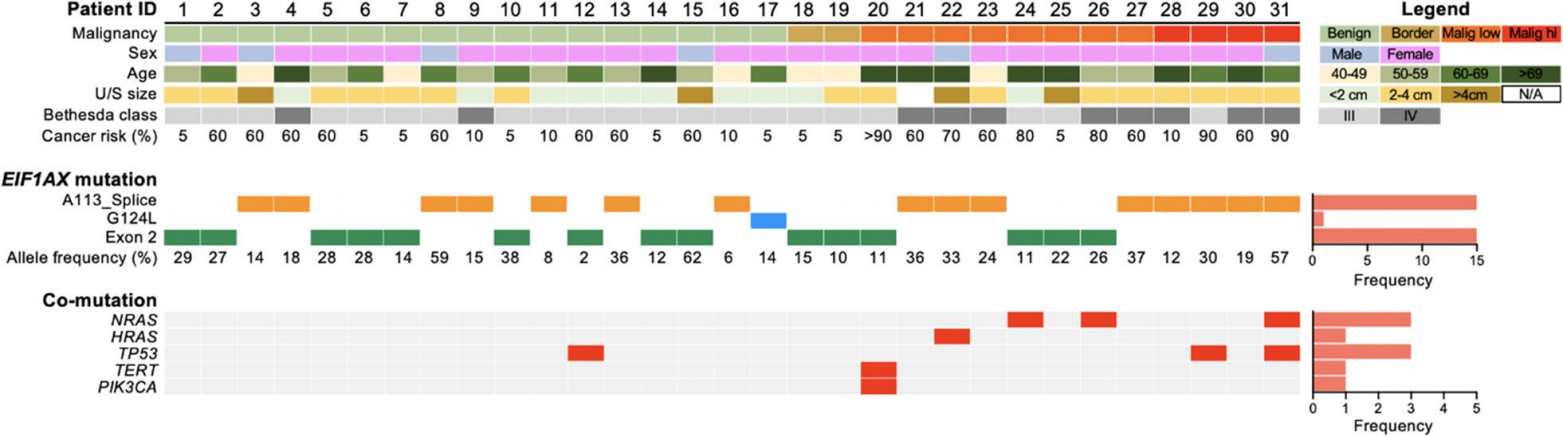
Overall mutation profile of patients with *EIF1AX* mutated tumors. The panels are aligned vertically by 31 patients included in the study. U/S: Ultrasound

**Fig. 2:**
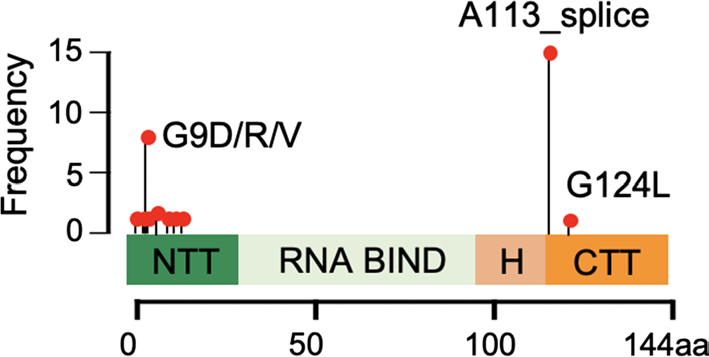
Frequency of *EIF1AX* mutation sites

Of the *EIF1AX* mutations, the most prevalent mutation was the A113_ splice site mutation (n = 15, 48%). Other than one G124L, also found in the C-terminal, all other mutations occurred in the N-terminal of the gene in exon 2 (Figs. 1 and 2). Some of these mutations have not been documented in previous literature (p.G6V, p.N11_E20dup, p.N17_K23dup, p.R13L, p.G8E, p.K16E).

**Fig. 3:**
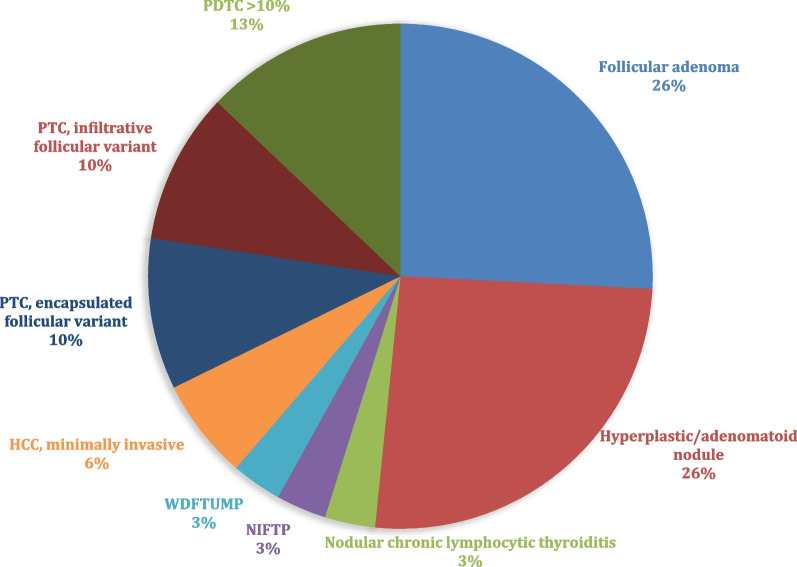
Frequency of histopathology diagnosis of *EIF1AX* mutated thyroid nodules. PDTC: Poorly Differentiated Thyroid Carcinoma; PTC: Papillary Thyroid Carcinoma; WDFTUMP: Well-differentiated follicular neoplasm of uncertain malignant potential; NIFTP: Noninvasive follicular thyroid neoplasm with papillary-like nuclear features; HCC: Hürthle cell (oncocytic) carcinoma

Of the 31 *EIF1AX* mutation samples, 17 (54.8%) were benign, 2 (6.5%) were borderline, and 12 (38.7%) were found to be malignant. Distribution and detailed histopathologic classification are presented in Fig. 3. There were 4 cases with a significant (> 10%) PDTC component (3 HCC and 1 PTC).

Amongst the 15 tumors with A113_splice mutations, 8 were malignant (53%). In comparison, out of the 16 tumors with non-A113_splice mutations, only 4 were malignant (25%). A113_splice mutation was not significantly associated with higher frequency of malignancy as compared to non-A113_splice mutations (Chi-Square: 2.620; df:1; *p* value: 0.106). When analyzing only malignant tumors (n = 12), all non-A113_splice mutation tumors were low-risk-malignancies (n = 4) whereas A113_splice mutation showed 4 aggressive malignancies and 4 low-risk-malignancies. This did not confer an increased chance of aggressive malignancy in A113_splice mutation (Fisher’s Exact Test, *p* value: 0.208).

Out of the 31 samples, 24 had singular mutations, that is, without any co-mutations. 12 samples were singular non-A113_splice mutations, and 12 were singular A113_splice mutations. Of the 12 A113_splice mutations, 5 were malignant and 7 were benign or borderline, compared to 12 non-A113_splice mutation in which only 1 was malignant. Statistically, singular A113_splice mutations did not show increased frequency of malignancy compared to singular non-A113_splice mutations (Chi-Square 3.556; df:1; *p* value: 0.059).

7 specimens harbored co-mutations, of which 6 were malignant (85.7%). These mutations included *NRAS (n* = 3*), HRAS (n* = 1*), TP53 (n* = 3*), TERT (n* = 1*), and PIK3CA (n* = 1*)*. The 4 sample with *RAS* co-mutations were all malignant. Of the 4 *RAS* mutations, 2 tumors had A113_splice, and 2 tumors had non-A113_splice mutations (p.R13P, p.G9D). Interestingly, one of the two tumors with A113_splice co-mutation with *RAS* had some degree of PDTC. The 2 *RAS* tumors co-mutated with non-A113_splice mutation were both PTC infiltrative follicular variant.

In order to get a better sense of the significance of these mutations (eg *RAS*) in the absence of *EIF1AX* co-mutation, we looked at all FNA samples that had molecular testing, including Bethesda 5 and 6 samples, and collected all non-*EIF1AX* mutations. 268 tumors with gene mutations other than *EIF1AX* were identified. Of these tumors, 239 (89.2%) with either BRAF, RAS, TERT or TP53 mutation were malignant. (Table 2) In our study, 85.7% of tumors with *EIF1AX* and these co-mutations were malignant. It is of great interest however, that *RAS* mutation on its own only represented 81.5% of malignancy, whereas 4 *RAS* tumors with *EIF1AX* co-mutations were all malignant.

**Table 2:** Tumors with *BRAF, RAS, TERT,* or *TP53* mutations without coexisting *EIF1AX* mutation

	Malignant	Borderline	Benign	Unknown/not reported
BRAF	129	0	1	2
RAS	106	12	12	1
TERT	3 (1 PDTC)	0	1	0
TP53	1 (1 PDTC)	0	0	0

## Discussion

To our knowledge, this is the largest cohort of thyroid FNA samples with indeterminate cytology (Bethesda III and IV) harboring *EIF1AX* mutations with surgical follow-up.

### Frequency of EIF1AX mutation in thyroid nodules

Amongst a total of 621 thyroid FNA specimens of indeterminate cytology with molecular testing, the frequency of the *EIF1AX* mutation was 4.99% which is similar to previously reported rates of 4.5% (14/904) and 4.2% (27/647) [2, 11]. As with previous studies, the most common mutation in the *EIF1AX* gene was the A113_splice site. In addition to previously described exon 2 mutations, we identified new exon 2 mutations not previously mentioned in the literature. One new mutation identified, the p.N17_K23dup, was associated with malignant histopathology (minimally invasive HCC).

### Association of EIF1AX mutation with malignancy

In our study, of the 31 Bethesda III and IV nodules with *EIF1AX* mutation, 17 were benign, 2 were borderline, and 12 were malignant on surgical follow-up, conferring an overall risk of malignancy (ROM) of 38.7% and a ROM or borderline of 45.2%.

Our results align with previous findings that *EIF1AX* mutations occur in both benign and malignant thyroid nodules, and that they also coexist with other driver mutations including *NRAS, HRAS, TP53, TERT,* and *PIK3CA* in a significant proportion of cases.

Though 8/12 (67%) of malignant nodules harbored the A113_splice mutation, it was not significantly associated with malignancy when compared to non-A113_splice mutations (*p* = 0.106). Moreover, all aggressive malignancies had the A113_splice mutation (n = 4), but the presence of the A113_splice *EIF1AX* mutation was not statistically associated with more aggressive malignancies, such as PDTC or ATC. Singular A113_splice mutations (without co-mutations), were not statistically associated with malignancy but did show a strong correlation with malignancy (*p* = 0.059). We highlight that out of the 12 singular non-A113_splice mutation tumors, 11 were of benign pathology. The statistical significance may not have been achieved due to small number of samples.

### Role of EIF1AX and co-mutations in malignancy risk stratification

When considering only cases with an *EIF1AX* mutation in isolation (i.e., no co-mutation), the ROM was 25% (6/24) and ROM or borderline was 33.3% (8/24). In the literature, isolated *EIF1AX* mutation is estimated to carry ~ 20–36.4% of ROM. In their recent study of 26 cases, Gargano et al. found a ROM of 36.4% (4/11) and a ROM or NIFTP of 54.5% (6/11) for cases with *EIF1AX* mutation alone and no other coexisting mutations [2, 11]. In their study, Karunamurthy et al. found *EIF1AX* mutations in 27/647 (4.2%) of indeterminate cytology samples, of which only 5 had surgical follow-up information (1 Encapsulated follicular variant PTC, 1 hyperplastic nodule, and 3 FA) yielding an estimated ROM of 20%. Therefore, the clinical value of finding an *EIF1AX* mutation in isolation in a thyroid nodule with indeterminate cytology (Bethesda III/IV) is limited as it does not change the ROM conferred by FNA significantly (~ 10–40% for Bethesda III/IV).

As the *EIF1AX* mutation alone is limited in bettering the malignancy risk stratification, co-mutation profile with *EIF1AX* plays a more integral role in the risk evaluation. In our study, the coexistence of *EIF1AX* mutations with other driver pathogenic mutations such as *RAS, TERT* and *TP53* conferred an 86% ROM (6 out of 7 cases). Similarly, Gargano et al. [11] found that *EIF1AX* mutation with *RAS* mutation (seen in 26.9% of their cases) conferred a 71.4% ROM (85.7% with NIFTP), while *EIF1AX* with any other mutation (eg., *TP53, TERT, RAS* + *TERT* or *TP53*, *BRAF* fusion, seen in 30.6% of their cases) conferred a 100% ROM. Similarly, Karunamurthy et al. found co-mutations in 3/11 cases (2 cases with only *NRAS* and one with *NRAS*, *TP53* and *TERT*), all of which were malignant neoplasms [2]. Therefore, surgical intervention with at least lobectomy should be considered for patients with such co-mutation profile.

Given the high allelic frequency of *EIF1AX* mutations in comparison to that of co-existing mutations, it has been suggested that *EIF1AX* mutations represent an early event, at least in some cases, that promotes initiation of the thyroid tumors and malignant transformation [11]. In other words, *EIF1AX* mutations alone are not sufficient for full transformation, but requires other mutations, particularly *RAS*, for progression to overt malignancy. Therefore, the clinical significance of finding an isolated EIF1AX mutation in a thyroid nodule, even though the immediate outcome is likely to be benign, is uncertain. Even nodules that histologically look like hyperplastic/adenomatoid nodules and were classified as such (8 cases in our series) can be clonal tumors.

The type of *EIF1AX* mutation also appears to render different outcomes with co-mutation (Table 3). Notably, the co-occurrence A113_splice mutation with *RAS* correlated with malignancy and aggressive tumor behavior in our study. All 4 samples with *RAS* co-mutations in our study were malignant. Moreover, one tumor with A113_splice and *RAS* mutation displayed PDTC characteristics. Therefore, we also confirm the existence of *RAS* mutation with *EIF1AX* and its association with more poorly differentiated malignancies [1, 2, 7, 12]. In contrast, exon 2 mutations without *RAS* mutation were only seen in benign, borderline and PTCs [2, 13].

**Table 3:** Compilation of malignancy risk stratification according to the EIF1AX mutation type and co-mutation profile from current study, Gargano et al. [11], and Karunamurthy et al. [2]

	Single mutation	Co-mutation
A113_Splice	12 Benign (57.1%) 1 Borderline (4.8%) 6 Malignant (28.6%) 2 Aggressive Malignant (9.5%)	14 Malignant (82.3%) 3 Aggressive Malignant (17.7%)
Non-A113_Splice	16 Benign (72.7%) 3 Borderline (13.6%) 3 Malignant (13.6%)	2 Benign (25%) 1 Borderline (12.5%) 5 Malignant (62.5%)

A113_splice mutation had higher rates of malignancy, especially when present with co-mutations. Co-mutations in this analysis include *RAS*, *TP53*, *TERT*, *YWHAG-BRAF* fusion, *PIK3CA*

### Histopathology of nodules harboring EIF1AX mutation

In the literature, the frequency of *EIF1AX* mutations in all PTCs is reported to be 1–2.5% [2, 4–6]. The majority of the studies found these tumors to be predominantly encapsulated follicular variant [1, 2, 11]. In our study, we had a similar proportion of encapsulated FVPTC and infiltrative FVPTC (10% each). In addition to FVPTCs, most other cases with *EIF1AX* mutation, both benign and malignant, were follicular phenotype (i.e., with a follicular growth pattern) including hyperplastic/adenomatoid nodules, FA, NIFTP, WDFT-UMP, and Hürthle cell neoplasms. Similarly, Gargano et al. found most of their *EIF1AX* mutated tumors to be follicular phenotype with or without papillary nuclear features and/or oncocytic features (FA, FTC, NIFTP, FVPTC, and Hürthle cell) [11]. These findings are to be expected since these neoplasms are the most common histologic follow-up for thyroid nodules classified as Bethesda III and IV on cytology. In addition, NIFTP, encapsulated and invasive FVPTC cannot be distinguished on cytology, as their distinction requires the histologic evaluation of the whole tumor.

In our study, a significant proportion (38.7%) of both benign and malignant nodules were oncocytic or had oncocytic features, including 8/12 malignancies and the four cases (13% of nodules) with a PDTC component. *EIF1AX* mutation was previously reported with a high incidence in HCC, highlighting the importance of this gene for this particular histopathology. In a comprehensive genomic characterization of 56 primary HCC tumors, Ganly et al. [14] identified *EIF1AX* mutations in 11% of cases, a frequency similarly seen in PDTC and ATC. The co-occurrence of *EIF1AX* and *RAS* mutations was not observed in their study. In a recent study where molecular testing was performed in 85 thyroid FNA specimens that were Bethesda IV-suspicious for a Hürthle cell neoplasm, *EIF1AX* mutations were found in 5 cases and, of those, three had surgical follow-up, two of which were HCC (including one with a co-*TERT* mutation) and one showed multinodular goiter on histologic evaluation [15]. Five other cases of HCC with *EIF1AX* mutation were reported previously. All were of A113_splice mutations, two of which had a co-mutation of *TP53* [11, 16, 17]. In contrast, Karunamurthy et al. did not find *EIF1AX* mutations in their study of 53 FTCs, including 22 HCCs.

### Study limitations

There are many limitations to our retrospective study. Firstly, the study was limited to Bethesda III/IV nodules with surgical follow-up. As a result, we were not able to report the frequency of *EIF1AX* mutations in specific types of thyroid neoplasms including conventional PTC or ATC that usually correlate with Bethesda V or VI categories. Secondly, the estimated ROM calculated in studies such as this one may overestimate the actual ROM, due to the impact of selection bias. Nodules that undergo surgical resection are more likely to have suspicious pre-operative clinico-radiological findings, increasing the likelihood of malignancy regardless of the FNA diagnosis and molecular result. We note, however, that while 55% of thyroid nodules within this study were benign at the time of surgery, their natural history, including the possible progression to a malignancy and/or the acquisition of a second mutation (eg., *TP53* or *TERT*) if they were not removed, is unknown. Indeed, *EIF1AX* mutations may represent an early event that promotes initiation of the thyroid tumors and malignant transformation in a subset of cases. In similar regards, we did not have long-term follow-up of the patients with malignancy. This is of particular importance as, *EIF1AX*-mutated PDTCs showed significantly shorter survival and were present in larger tumors when compared to wild type *EIF1AX* PDTCs [7]. Finally, although our study had the largest collection of *EIF1AX* mutations with histopathology correlation, the sample size remains small and necessitate further meta-analyses.

## Conclusion

Our study reports the largest cohort of *EIF1AX* mutations in Bethesda III/IV FNA samples with surgical follow-up to our knowledge. The presence of the *EIF1AX* mutation in Bethesda III and IV thyroid nodules confers a 45.2% ROM including borderline tumors (ROM only = 38.7%). However, the coexistence of *EIF1AX* mutations with other driver pathogenic mutations such as *RAS, TERT* and *TP53* conferred a much higher ROM (86%). A third of the malignancies were aggressive, harboring a significant component of PDTC. While 55% of thyroid nodules within this study were benign at the time of surgery, the natural history of these nodules (i.e., possible progression to a malignancy if they were not removed) is unknown. These finding can further aid in clinical decision making for patients with thyroid nodules.

## Data Availability

The datasets generated and/or analyzed during the current study are not publicly available due the privacy of the patients (eg hospital record number) but are available from the corresponding author on reasonable request.

## Abbreviations

ATC: Anaplastic thyroid carcinoma
AUS: Atypia of undetermined significance
*EIF1AX*: Eukaryotic translation initiation factor 1A, X-linked
FA: Follicular adenoma
FLUS: Follicular lesion of undetermined significance
FN: Follicular neoplasm
FNA: Fine needle aspirate
FTC: Follicular thyroid carcinoma
HTC: Hürthle cell carcinoma
NH: Nodular hyperplasia
NIFTP: Non-invasive follicular thyroid neoplasm with papillary like nuclear features
PDTC: Poorly differentiated thyroid carcinoma
PTC: Papillary thyroid carcinoma
ROM: Risk of malignancy
TCGA: The cancer genome atlas
WDFTUMP: Well-differentiated follicular neoplasm of uncertain malignant potential
U/S: Ultrasound
